# Supplementary material for: Observation of spin-space quantum transport induced by an atomic quantum point contact
Source: Nat Commun. 2021 Nov 18;12:6724. doi: 10.1038/s41467-021-27011-2 (PMC8602744; doi:10.1038/s41467-021-27011-2)
Supplement: Supplementary file 1 — Supplementary Information [file 41467_2021_27011_MOESM1_ESM.pdf]

Supplementary Information

Koki Ono,[1,*] Toshiya Higomoto,[1] Yugo Saito,[1] Shun Uchino,[2] Yusuke Nishida,[3] and Yoshiro Takahashi[1]

[1] *Department of Physics, Graduate School of Science, Kyoto University, Kyoto 606-8502, Japan*
[2] *Advanced Science Research Center, Japan Atomic Energy Agency, Tokai, Ibaraki 319-1195, Japan*
[3] *Department of Physics, Tokyo Institute of Technology, Ookayama, Meguro, Tokyo 152-8551, Japan*

(Dated: July 30, 2021)

SUPPLEMENTARY NOTE 1: CALCULATION OF SCATTERING PHASE SHIFT

First, we consider the quasi 1D system, where the $|g\rangle$ and $|e\rangle$ atoms are radially confined by the 2D state-independent optical lattice and calculate the effective 1D scattering length [1]. Next, we consider the scattering problem in the effective 1D system, where the $|e\rangle$ atom is localized by the 1D near-resonant optical lattice and calculate the scattering phase shift using the effective 1D scattering length [2].

A. Effective 1D scattering length

We consider the two-channel model consisting of the open channel $|o\rangle \equiv |g \Uparrow; e \Downarrow\rangle$ and the closed channel $|c\rangle \equiv |g \Downarrow; e \Uparrow\rangle$. The scattering problem in the two-channel model is studied in Supplementary Ref. [1], and we calculate the effective 1D scattering length $a_{1\mathrm{D}}$ based on our experimental parameters. In the case of the two-terminal experiment, $|\Uparrow\rangle = |m_F = +5/2\rangle$ or $|m_F = +1/2\rangle$, and $|\Downarrow\rangle = |m_F = -5/2\rangle$. The non-interacting Hamiltonian in the relative coordinates $\hat{\mathcal{H}}_0$ is given by

$$\hat{\mathcal{H}}_0 = \begin{pmatrix} \hat{H}_z + \hat{H}_\perp & 0 \\ 0 & \hat{H}_z + \hat{H}_\perp + \delta(B) \end{pmatrix}, \tag{1}$$

where $\delta(B) = h\delta\mu B > 0$ denotes the Zeeman energy difference between the two channels with $\delta\mu = 110.72(51)\Delta m_F$ Hz/Gauss [3]. Here $\hat{H}_z + \hat{H}_\perp$ represents the free-particle Hamiltonian:

$$\hat{H}_z = -\frac{\hbar^2}{2\mu}\frac{\partial^2}{\partial z^2}, \tag{2}$$

$$\hat{H}_\perp = -\frac{\hbar^2}{2\mu}\left(\frac{\partial^2}{\partial \rho^2} + \frac{1}{\rho}\frac{\partial}{\partial \rho} - \frac{m_z^2}{\rho^2}\right) + \frac{1}{2}\mu\omega_\perp^2\rho^2. \tag{3}$$

Here $\mu = m/2$ is the reduced mass, and $\omega_\perp = 2\pi \times 22.0$ kHz is the radial trap frequency of the tube potential, and $\hat{H}_z$ and $\hat{H}_\perp$ satisfy the following Schrödinger equations:

$$\hat{H}_z \xi_k(z) = \frac{\hbar^2 k^2}{2\mu}\xi_k(z), \tag{4}$$

$$\hat{H}_\perp \chi_{n_\perp, m_z}(\rho) = (n_\perp + 1)\hbar\omega_\perp \chi_{n_\perp, m_z}(\rho), \tag{5}$$

where $\xi_k(z) = e^{ikz}/\sqrt{2\pi}$ represents the plane wave with the wave number k, and $\chi_{n_\perp,m_z}(\rho)$ is the eigenfunction of $\hat{H}_\perp$, with $m_z = 0, \pm1, \pm2, \ldots$ and $n_\perp = |m_z|, |m_z|+2, |m_z|+4, \ldots$. The interaction Hamiltonian $\hat{\mathcal{V}}$ is given by the 3D Fermi pseudopotential:

$$\hat{\mathcal{V}} = \frac{2\pi\hbar^2}{\mu} A_{3\mathrm{D}} \delta(\boldsymbol{r}) \left(\frac{\partial}{\partial r} r \,\cdot \right), \tag{6}$$

where

$$A_{3\mathrm{D}} = \begin{pmatrix} a_{\mathrm{d}} & a_{\mathrm{ex}} \\ a_{\mathrm{ex}} & a_{\mathrm{d}} \end{pmatrix}. \tag{7}$$

Here $a_{\mathrm{d}} = (a_{eg}^+ + a_{eg}^-)/2$ and $a_{\mathrm{ex}} = (a_{eg}^+ - a_{eg}^-)/2$, and $a_{eg}^+ = 1878(37)a_{\mathrm{B}}$ and $a_{eg}^- = 220(2)a_{\mathrm{B}}$ (a_{B}: Bohr radius) correspond to the spin-singlet and spin-triplet interorbital scattering lengths, respectively [4].

In order to solve the scattering problem, the incident wave $\boldsymbol{\Psi}_0(\rho, z) = \xi_k(z)\chi_{0,0}(\rho)\,|o\rangle$, with energy $E = \hbar^2k^2/(2\mu) + \hbar\omega_\perp$, is considered. Here we assume the low-energy scattering $\hbar^2k^2/(2\mu) < \hbar\omega_\perp, \delta(B)$. The scattering wave function $\boldsymbol{\Psi}(\rho, z)$ is obtained by solving the Lippmann-Schwinger equation:

$$\boldsymbol{\Psi}(\rho, z) = \boldsymbol{\Psi}_0(\rho, z) + \frac{2\pi\hbar^2}{\mu} \begin{pmatrix} G_E(\rho, z; 0, 0) & 0 \\ 0 & G_{E-\delta(B)}(\rho, z; 0, 0) \end{pmatrix} A_{3\mathrm{D}} \boldsymbol{\eta}, \tag{8}$$

where $G(\rho, z; \rho', z')$ is the free Green's function, and $\boldsymbol{\eta}$ is the regularized scattering wave function, defined as

$$\boldsymbol{\eta} = \begin{pmatrix} \eta_{\mathrm{oc}} \\ \eta_{\mathrm{cc}} \end{pmatrix} = \frac{\partial}{\partial z} \{ z \boldsymbol{\Psi}(0, z) \}_{z \to 0+}. \tag{9}$$

The equation with respect to $\boldsymbol{\eta}$ is obtained by substituting Supplementary Equation (8) into Supplementary Equation (9):

$$\boldsymbol{\eta} = \boldsymbol{\Psi}_0(0, 0) + \frac{2\pi\hbar^2}{\mu} \frac{\partial}{\partial z} \left\{ z \begin{pmatrix} G_E(0, z; 0, 0) & 0 \\ 0 & G_{E-\delta(B)}(0, z; 0, 0) \end{pmatrix} A_{3\mathrm{D}} \boldsymbol{\eta} \right\}_{z \to 0+}. \tag{10}$$

Here $G_E(0, z; 0, 0)$ and $G_{E-\delta(B)}(0, z; 0, 0)$ are calculated as follows:

$$G_E(0, z; 0, 0) = -\frac{\mu}{\pi a_\perp^2 \hbar^2} \left(i\frac{e^{ik|z|}}{k} + \sum_{n=1,2,\ldots} \frac{e^{-\kappa_n |z|}}{\kappa_n} \right), \tag{11}$$

$$G_{E-\delta(B)}(0, z; 0, 0) = -\frac{\mu}{\pi a_\perp^2 \hbar^2} \sum_{n=0,1,2,\ldots} \frac{e^{-\lambda_n(B)|z|}}{\lambda_n(B)}, \tag{12}$$

where $a_\perp = \sqrt{\hbar/(\mu\omega_\perp)} = 1377a_{\mathrm{B}}$ denotes the oscillator length. Here κ_n and $\lambda_n(B)$ are given by

$$\kappa_n = \sqrt{\left(\frac{2}{a_\perp}\right)^2 n - k^2}, \tag{13}$$

$$\lambda_n(B) = \sqrt{\left(\frac{2}{a_\perp}\right)^2 n + \gamma(B)^2 - k^2}, \tag{14}$$

where $\gamma(B) = \sqrt{2\mu\delta(B)}/\hbar$.

We obtain the scattering wave function of the open channel in the limit $|z| \to \infty$:

$$\psi_{\rm oc}(\rho, |z| \to \infty) \to \frac{1}{\sqrt{2\pi}}\{e^{ikz} + f(k)e^{ik|z|}\}\chi_{00}(\rho), \tag{15}$$

where $f(k)$ represents the scattering amplitude given by

$$f(k) = -\frac{2\sqrt{2}\pi i}{a_\perp k}(a_{\rm d}\eta_{\rm oc} + a_{\rm ex}\eta_{\rm cc}). \tag{16}$$

The regularized scattering wave function $\boldsymbol{\eta}$ is calculated as follows:

$$\boldsymbol{\eta} = \frac{1}{\sqrt{2}\pi a_\perp}\left|o\right\rangle - MA_{\rm 3D}\boldsymbol{\eta} = \frac{1}{\sqrt{2}\pi a_\perp}(\mathbf{1}_2 + MA_{\rm 3D})^{-1}\left|o\right\rangle, \tag{17}$$

where the matrix M is defined as

$$M = {\rm diag}\left[\frac{2i}{a_\perp^2 k} + \frac{1}{a_\perp}\zeta\left(\frac{1}{2}, 1 - \left(\frac{ka_\perp}{2}\right)^2\right),\ \frac{1}{a_\perp}\zeta\left(\frac{1}{2}, \left(\frac{\lambda_0(B)a_\perp}{2}\right)^2\right)\right]. \tag{18}$$

Here $\zeta(1/2, z)$ is the Hurwitz zeta function identical to $\mathcal{L}(z-1)$ introduced in Supplementary Ref. [5]. The scattering amplitude $f(k)$ can be expressed as

$$f(k) = -\frac{2i}{a_\perp^2 k}\left\langle o\right|(A_{\rm 3D}^{-1} + M)^{-1}\left|o\right\rangle. \tag{19}$$

Thus the effective 1D scattering length $a_{\rm 1D}$ is obtained as

$$\begin{aligned} a_{\rm 1D} &= \lim_{k\to+0} -\frac{1 + f(k)^{-1}}{ik} \\ &= -\frac{a_\perp}{2}\left(\frac{a_\perp}{a_0} + \zeta\left(\frac{1}{2}, 1\right)\right) + \frac{a_\perp}{2}\frac{(a_\perp/a_1)^2}{a_\perp/a_0 + \zeta(1/2, (\gamma(B)a_\perp)^2/4)}, \end{aligned} \tag{20}$$

where $a_0 = (a_{\rm d}^2 - a_{\rm ex}^2)/a_{\rm d}$ and $a_1 = (a_{\rm d}^2 - a_{\rm ex}^2)/a_{\rm ex}$.

Supplementary Figure 1 shows the effective 1D scattering length as a function of the magnetic field for the parameter set relevant to our experiment. In particular, we obtain $a_{\rm 1D}/a_{\rm B} = 515$ ($\Delta m_F = 5$), 165 ($\Delta m_F = 3$) at 45 Gauss and $a_{\rm 1D}/a_{\rm B} = 2667$ ($\Delta m_F = 5$), 1212 ($\Delta m_F = 3$) at 135 Gauss.

B. Scattering problem in (0+1)D system

Here we consider a scattering problem in a (0+1)D system, where the $|g\rangle$ atom and the $|e\rangle$ atom are itinerant and localized, respectively. This problem is studied in Supplementary Ref. [2], and we calculate the scattering phase shifts based on our experimental parameters. The non-interacting Hamiltonian $\hat{H}_0$ is given by

$$\hat{H}_0 = \hat{H}_g + \hat{H}_e = -\frac{\hbar^2}{2m}\frac{\partial^2}{\partial z_g^2} - \frac{\hbar^2}{2m}\frac{\partial^2}{\partial z_e^2} + \frac{1}{2}m\omega_z^2 z_e^2, \tag{21}$$

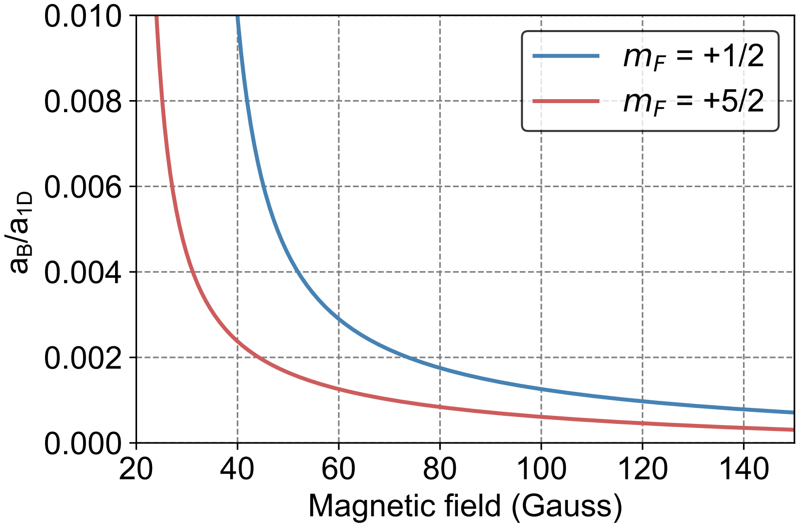

Supplementary Figure 1: **Magnetic-field dependence of the effective 1D scattering length.** The open channel is defined as $|o\rangle = |g, +5/2; e, -5/2\rangle$ (a red line) or $|o\rangle = |g, +1/2; e, -5/2\rangle$ (a blue line).

where $z_{g(e)}$ denotes the z coordinate of the $|g\rangle(|e\rangle)$ atom, and $\omega_z = 2\pi \times 24.4$ kHz is the trap frequency of the near-resonant optical lattice for the $|e\rangle$ atom. We assume that the confinement of the near-resonant optical lattice is negligible for the $|g\rangle$ atom. The Hamiltonians $\hat{H}_g$ and $\hat{H}_e$ satisfy the following Schrödinger equations:

$$\hat{H}_g \xi_k(z_g) = \frac{\hbar^2 k^2}{2m} \xi_k(z_g), \tag{22}$$

$$\hat{H}_e \phi_{n_z}(z_e) = \left(n_z + \frac{1}{2} \hbar\omega_z \right) \phi_{n_z}(z_e). \tag{23}$$

The interaction part is given by the effective 1D potential:

$$\hat{V}_{1\mathrm{D}}(z_e, z_g) = -\frac{2\hbar^2}{m a_{1\mathrm{D}}} \delta(z_e - z_g). \tag{24}$$

The incident wave $\psi_0(z_e, z_g) = \xi_k(z_g)\phi_0(z_e)$, with energy $E = \hbar^2 k^2/2m + \hbar\omega_z/2$, is considered, and the scattering wavefunction $\psi(z_e, z_g)$ satisfy the Lippmann-Schwinger equation as follows:

$$\psi(z_e, z_g) = \psi_0(z_e, z_g) - \frac{2\hbar^2}{m a_{1\mathrm{D}}} \int_{-\infty}^{+\infty} \mathrm{d}z' g_E(z_e, z_g; z', z') \psi(z', z'), \tag{25}$$

where $g_E(z_e, z_g; z', z')$ represents the two-body free Green's function. Here we assume the low-energy scattering $\hbar^2 k^2/2m < \hbar\omega_z$. Thus the Green's function is expressed as

$$g_E(z_e, z_g; z', z') = -\frac{im}{\hbar^2} \frac{e^{ik|z_g - z'|}}{k} \phi_0(z_e)\phi_0^*(z') - \frac{m}{\hbar^2} \sum_{n=1,2,\ldots} \frac{e^{-\kappa_n |z_g - z'|}}{\kappa_n} \phi_n(z_e)\phi_n^*(z'), \tag{26}$$

where $\kappa_n = \sqrt{2n/a_z^2 - k^2}$, with a_z being the oscillator length $a_z = \sqrt{\hbar/(m\omega_z)} = 925a_{\rm B}$.

We obtain the scattering wave function in the limit $|z_g| \to \infty$:

$$\psi(z_e, |z_g| \to \infty) \to \frac{1}{\sqrt{2\pi}} \left[e^{ikz_g} + \{f_0(k) + \mathrm{sgn}(z_g) f_1(k)\} e^{ik|z_g|} \right] \phi_0(z_e). \tag{27}$$

The scattering amplitudes $f_0(k)$ and $f_1(k)$ are expressed as

$$f_0(k) = \frac{2\sqrt{2\pi}i}{ka_{\rm 1D}} \int_{-\infty}^{+\infty} \mathrm{d}z' \cos kz' \phi_0^*(z') \psi(z', z'), \tag{28}$$

$$f_1(k) = \frac{2\sqrt{2\pi}}{ka_{\rm 1D}} \int_{-\infty}^{+\infty} \mathrm{d}z' \sin kz' \phi_0^*(z') \psi(z', z'). \tag{29}$$

The function $\psi(z, z)$ satisfies the following integral equation:

$$\psi(z, z) = \psi_0(z, z) - \frac{2\hbar^2}{ma_{\rm 1D}} \int_{-\infty}^{+\infty} \mathrm{d}z' g_E(z, z; z', z') \psi(z', z'). \tag{30}$$

On the other hand, the scattering amplitude can be expressed with the scattering phase shift $\delta_l(\varepsilon)$:

$$f_l(k) = -\frac{1}{1 + i \cot \delta_l(\hbar^2 k^2/2m)}. \tag{31}$$

We calculate the scattering amplitudes $f_0(k)$ and $f_1(k)$ by solving Supplementary Equation (30) numerically. Supplementary Figure 2a-b show the scattering phase shifts of the atom in the $|\uparrow (\downarrow)\rangle$ state as functions of ε, where the $|\uparrow\rangle$ and $|\downarrow\rangle$ states correspond to the $|g\rangle\,|m_F = +5/2\rangle$ and $|g\rangle\,|m_F = +1/2\rangle$ states, respectively. We find that $\delta_{l\uparrow} - \delta_{l\downarrow}$ for $l = 1$ at 135 Gauss is larger than that at 45 Gauss, which accounts for the faster transport dynamics observed in a magnetic field of 135 Gauss (see Fig. 5 in the main text).

SUPPLEMENTARY NOTE 2: CALCULATION OF TRANSPORT DYNAMICS

As mentioned in the main text, the time derivative of the atom-number difference ΔN is the current, determined by the Landauer-Büttiker formula (see Equation (3) in the main text). The fugacity $e^{\beta\mu_{+(-)}}$, with $\mu_{+(-)}$ being the chemical potential of the atoms in the $|+(-)\rangle$ state, is expressed using Equation (11) in the main text:

$$e^{\beta\mu_+} = e^{\beta\hbar\omega_{\rm trap} N_+} - 1 = e^{\beta\hbar\omega_{\rm trap}(N+\Delta N)/2} - 1, \tag{32}$$

$$e^{\beta\mu_-} = e^{\beta\hbar\omega_{\rm trap} N_-} - 1 = e^{\beta\hbar\omega_{\rm trap}(N-\Delta N)/2} - 1. \tag{33}$$

Thus the current is expressed in terms of ΔN assuming the total number of atoms N is conserved, and the transport dynamics is given by the first order differential equation of ΔN, which can be solved numerically. In order to perform the numerical calculation of the transport dynamics, we consider the spatial inhomogeneity of the atom numbers in the tube potentials, as shown in Equation (6) in the main text. Supplementary Figure 3 shows the calculated transport dynamics in a magnetic field of 45 Gauss.

---

* Electronic address: koukiono3@yagura.scphys.kyoto-u.ac.jp

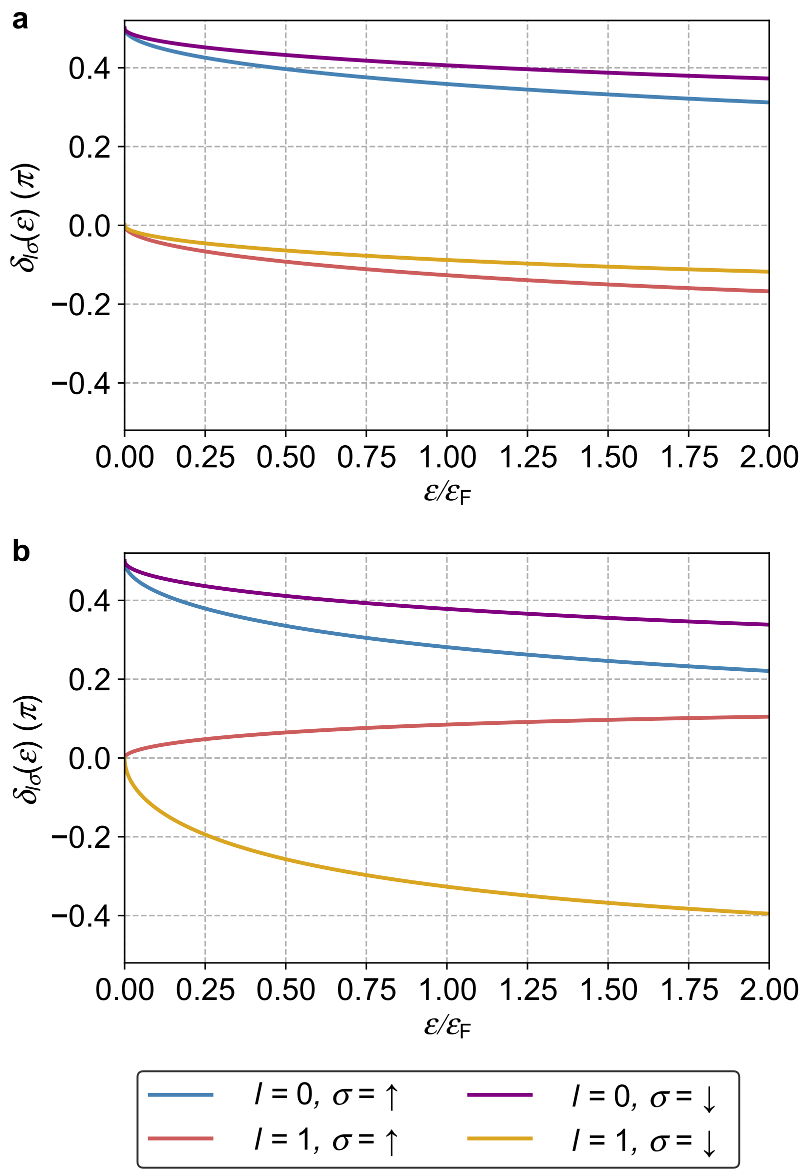

Supplementary Figure 2: **Energy dependence of scattering phase shifts.** **a**. 45 Gauss. **b**. 135 Gauss. Here $\varepsilon_{\mathrm{F}} = h \times 2$ kHz denotes the estimated Fermi energy in the central tube.

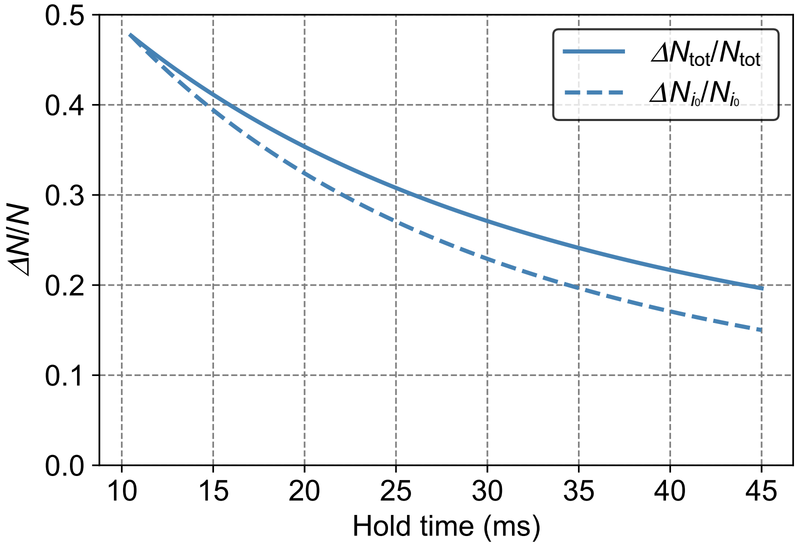

Supplementary Figure 3: **Calculation of transport dynamics in a magnetic field of 45 Gauss.** An index i_0 corresponds to the index of the central tube. The solid line is the same as the dashed line shown in Fig. 2c in the main text.

SUPPLEMENTARY REFERENCES

[1] Zhang, R. *et al.* Kondo effect in alkaline-earth-metal atomic gases with confinement-induced resonances. *Phys. Rev. A* **93**, 043601 (2016).
[2] Cheng, Y., Zhang, R., Zhang, P. & Zhai, H. Enhancing kondo coupling in alkaline-earth-metal atomic gases with confinement-induced resonances in mixed dimensions. *Phys. Rev. A* **96**, 063605 (2017).
[3] Darkwah Oppong, N. *et al.* Observation of coherent multiorbital polarons in a two-dimensional fermi gas. *Phys. Rev. Lett.* **122**, 193604 (2019).
[4] Höfer, M. *et al.* Observation of an orbital interaction-induced feshbach resonance in ^{173}Yb. *Phys. Rev. Lett.* **115**, 265302 (2015).
[5] Olshanii, M. Atomic scattering in the presence of an external confinement and a gas of impenetrable bosons. *Phys. Rev. Lett.* **81**, 938–941 (1998).
